# Dental Patents

**Published:** 1860-06

**Authors:** 


### DENTAL PATENTS.

MESSRS. EDITORS :—In the January number of the *N. Y. Dental Journal*, I find a communication over the signature of B. W. Franklin, which, I presume, is the long promised article which was to give me fits!! greatly to gratify the local and traveling agents of the American Hard Rubber Company and which they have since been using to still further intimidate the profession into the purchase of their *very* "liberal rights." I feel very badly under the influence of these unnatural *spasms!* but as Mr. F. is probably actuated by the same principles that governed a certain QUACK DOCTOR, whose first effort in all cases was to throw his patients into fits because he was "h—l on fits;" I think I may hope for speedy recovery.

My dear Mr. Franklin takes exception to a brief "business notice" in the January number of the *Dental Register*, and lest the profession should not be aware that I am proprietor, he repeats it in *Itatics* some half dozen times for which I thank him.

Mr. F. seems greatly concerned lest the *Register* should lose the respect of its subscribers and the profession. What disinterested kindness! He also asserts that the respectable portion of the profession will not sympathize with me in my "interested opposition to all dental patents." If he offers himself as a specimen, I hope not. If Mr. F. or any one would inform me wherein my interests in opposing dental patents exist I would feel greatly obliged to them, for thus far it has been to me only a great sacrifice of valuable time and expenditure of money for printing, postage, and to investigate the claims of the few dental patents which I have opposed.

But I ask no sympathy. What I have done has been at *my own expense;* the course I have pursued has been on my own resposibility. After having satisfied myself of the correctness of my position, and feeling my obligations to the profession, I pursue that course which conscience dictates regardless of whom it pleases or whom it displeases, whether in opposition to some upstart impostor, or a boastful monied monopoly. Others may whine like blind puppies, Mr. Franklin particularly plaintive and low if he chooses, but for my part I appeal to no man's lachrymose propensities, but I *challenge* the respect of all *men* and I know that the *unsought* judgment of the profession coincides with my view of dental patents,—and especially cautious should we be of indefinite ones.

As regards the *Dental Register* I have the satisfaction of knowing, and I think I can prove beyond cavil, that it is in better condition now than ever before. It is edited better, printed better, and *pays better* than any other dental journal that I know of, and it is the only one in the United States which stands up fully to its prospectus and claims and receives payment INVARIABLY IN ADVANCE.

The readers of the N. Y. *Dental Journal* must hold its editors and publishers in very light esteem if they are willing to condemn me for being *proprietor* of the *Dental Register*, as their correspondent would fain have them to do, nor did I imagine that by having a *Dental Depot* I became any the more reprehensible on general principles, though I can well conceive how particular circumstances may alter cases, for is not Mr. Franklin *agent* of the A. H. R. Co. at a salary of $2,000 a year, with abundance of leisure to discuss abolitionism and its kindred vagaries? Does this make him so zealous and so brave that he must fight his own shadow to make fair weather with his masters? Why did not Mr. Franklin fairly and frankly meet the issue and state plainly and precisely what the Goodyear patent is? what the scope as well as the limit of their rights to the manufacture of materials as well as the extent and boundary of their rights to the vulcanizing or curing process? It is a well established fact that *no one can patent a principle.* What then is the formula by which they manufacture material? is it four ounces of sulphur to one pound of gum, or is it a pound of sulphur to a pound of gum, or is it a *secret?* If he had done this he would be entitled to the respect of the profession for fairness and honesty at least, even if he should not sell so many rights.

With equal adroitness the agent dodges the question regarding the decision against Roberts, and questions my veracity in regard to the urgency of the A. H. R. Co. to sell rights, and immediately after states the fact that threatening circulars were issued to the profession. What is to be thought of the veracity of one who so completely contradicts himself in two consecutive sentences? It may be about as reliable as his published experiments on the strength of metals and other materials used for dental plates.

Why did he not state that the case was contested to the end, that there was no compromise and that Roberts received no benefits for permitting injunction to be entered up against him? This would have been satisfactory, but he contents himself with the statement that "suits were commenced against Roberts, he employed able counsel and the court granted permanent injunction. Whether the case was 'manfully contested' I leave for others to determine." He leaves it for others to determine who have no opportunity to obtain the facts, because those in possession of them seem to be interested in withholding them. What sort of truth this implies will appear hereafter. I will here reply to Mr. Franklin's inquiry as to who the N. Y. correspondent of the *Register* was, that by application to the editors, Drs. Taft and Watt, he can ascertain. He ought to have known this much of the rules governing the press without being told especially so,—as it is known that the editors of the N. Y. *Dental Journal* and the agent of the A. H. R. Co. occupy rooms jointly at 640 Broadway, New York.

Mr. Franklin reiterates that Mr. Goodyear gave notice again and again, to the profession that if they used the vulcanite process, without a license from him, they would be prosecuted to the full extent of the law. Why these repeated and unnecessary threats? Is it, to say the least, courteous to the profession to cry them continually, stop thief! stop thief!! Either as before remarked this monopoly is uncertain in its pretentions, or it treats the profession as a band of dishonest men and thieves, through its mouth piece, (whether self-constituted or not) Mr. Franklin.

In justice to the A. H. R. Co., I must say that the position which they occupied with the profession was very different before this speciality was placed in charge of Mr. Franklin from what it has been since, inasmuch as he has destroyed the feeling of satisfaction with which the new improvement was anticipated.

The Dental Profession is composed chiefly of high-toned honorable gentlemen, who will not tamely submit to be treated like thieves, or driven like dogs, for, unfortunately, Mr. Franklin has gauged the profession by his own narrow contracted views, and, although I do not think that either Mr

Goodyear or the A. H. R. Co., would voluntarily take the position they now occupy, they cannot well avoid the responsibility of their agent, although they may say to the profession as did Macbeth to Banquo's Ghost, "Shake not thy gory locks at me, thou canst not say I did it."

Mr. Franklin, as well as the profession, has had ample opportunity to know my position on Dental Patents. I am not opposed to *all* Dental Patents, nor have I knowingly invaded the rights of any, but I am opposed to such patents as have not a valid claim as well as to those endeavoring to cover under a valid claim that which their patent does not guarantee to them. I am also opposed to the *policy* of professional patenting, but this matter rests with the profession and not me to regulate.

The insinuation cast upon my personal honesty, with which Mr. Franklin fitly takes leave of his readers, I can well afford to overlook; feeling perfect assurance and security in the rectitude of my own thoughts and intentions—I shall issue no intimidating circulars to enforce an acknowledgement thereof. He gives some good advice and informs us that *he has* embraced the idea that "honesty is the best policy," I would be glad to know at what particular period this occurred, as I doubt whether his most intimate friends have discovered the *change.* JOHN T. TOLAND.

The foregoing article was intended for the N. Y. Dental Journal for April, but as neither the Journal nor article have made their appearance, I have chosen this as the medium for my reply.

In the "Business Notices" of the present number of the Register will be found some additional information in regard to this much talked of subject and process.

